# Margins of adaptation at the desert frontier: genetic responses of *Ammopiptanthus mongolicus* in arid northwestern China

**DOI:** 10.1186/s40529-025-00476-w

**Published:** 2025-09-29

**Authors:** Yong-Zhi Yang, De-Ming Gao, Pei-Wei Sun, Chong-Yi Ke, Qihui Fong, Min-Xin Luo, Run-Hong Gao, Pei-Chun Liao

**Affiliations:** 1 College of Forestry, Inner Mongolia Agricultural University, Huhhot, 010019 China; 2 Department of Ecology and Evolutionary Biology, Yale University, New Haven, Connecticut 06511 USA; 3 Department of Life Science, National Taiwan Normal University, No. 88 Ting-Chow Rd., Sec. 4, Taipei, 116 Taiwan; 4 Department of Ecology and Evolutionary Biology, University of Michigan, Ann Arbor, MI 48109-1085 USA

**Keywords:** Adaptation, *Ammopiptanthus mongolicus*, Arid environments, Climate change, Conservation genetics, Desert ecosystems, Drought tolerance, Marginal populations

## Abstract

**Background:**

Understanding plant adaptation to extreme environments is crucial for conservation and evolutionary biology. *Ammopiptanthus mongolicus*, a drought-resistant evergreen shrub native to northwestern China, provides an excellent model for studying genetic and ecological responses to arid conditions. Climatic fluctuations, especially during the Quaternary, have shaped its distribution and genetic diversity, influencing its ability to survive in desert environments. However, the mechanisms underlying its adaptation remain insufficiently explored.

**Main body:**

We synthesize findings from previous genomic, ecological, and biogeographical studies to evaluate the adaptive mechanisms of *A. mongolicus* and assess the conservation implications for desert plant populations. Northwestern China encompasses vast arid regions characterized by extreme environmental conditions, including low precipitation, high evaporation rates, and significant temperature fluctuations. The uplift of the Qinghai-Tibet Plateau increased aridity by blocking moist air, leading to the transformation of humid forests into drought-resistant deserts. *Ammopiptanthus mongolicus*, a broad-leaved evergreen shrub, serves as a model for studying plant adaptation to arid environments. Genomic studies have identified several genes and pathways associated with drought and cold adaptation in this species. Core populations of *A. mongolicus* inhabit stable environments and exhibit high genetic diversity, whereas marginal populations endure extreme conditions and show strong local adaptations and distinct genetic traits. In this review, we hypothesize that the geographical distribution of core and peripheral populations may shift in response to future climate change, with peripheral populations potentially serving as sources of adaptive alleles for extreme climatic conditions.

**Conclusions:**

Marginal populations of *A. mongolicus* are essential reservoirs of adaptive traits, providing genetic resources for coping with environmental stressors such as drought and cold. However, they face a higher risk of local extinction due to genetic load and habitat fragmentation. Gene flow between core and marginal populations may be crucial for maintaining genetic diversity and adaptive potential. Conservation strategies should prioritize protecting marginal populations to reduce genetic load, enhance resilience, and preserve genetic diversity in response to intensifying climate change.

Understanding plant adaptation to extreme environments is crucial for conservation and evolutionary biology. In the past, global climatic fluctuations have had lasting impacts on species’ genetic variation, distribution, adaptation, and even speciation. For example, climate changes during the Quaternary period led to repeated contractions and expansions of species distributions, resulting in significant diversification, particularly in temperate regions of the Northern Hemisphere (Hewitt 2000; Petit et al. 2003). Aridification specifically facilitated the diversification and speciation of desert plants (Hewitt 2000, 1996, 2004), though its specific role in species formation remains unclear (Futuyma 2010; Willis et al. 2004), especially in northwestern China. These past climate changes have important effects on current adaptations and provide examples of the possible effects of future climate changes, both of which have important implications for conservation efforts.

The biogeographic and evolutionary history of northwestern China’s deserts provides essential context for interpreting the current genetic structure and adaptive strategies of desert plants. Repeated Quaternary cycles of aridification and desert expansion not only reshaped species distributions but also created ecological and genetic conditions under which marginal and core populations emerged (Yang et al. 2022a). These historical processes contributed to the accumulation of rare alleles, founder effects, and local adaptations that are now critical for population persistence in extreme environments (Jiang et al. 2019). Furthermore, these genetic outcomes provide basic information to infer the future evolutionary trajectories of different populations. We hypothesize that core and peripheral populations in deserts, which have experienced distinct evolutionary histories, will respond differently and alter their population interactions under intensified future climate change. By first examining these biogeographic patterns and evolutionary drivers, we establish a framework for understanding the genetic and ecological differentiation of desert species, such as *Ammopiptanthus mongolicus*, and their contrasting roles in resilience to future climate change. We highlight how past climatic changes can influence plant biogeography and adaptation in general and then more specifically in the less well-studied northwestern China climate. Then we use *A. mongolicus* as a case study to explore how past climates have impacted desert adaptation and what this means for conservation under future climate changes.

## Quaternary climatic oscillations and desert plant diversification

### Desert expansion and its role in speciation of drought-tolerant plants

**Fig. 1:**
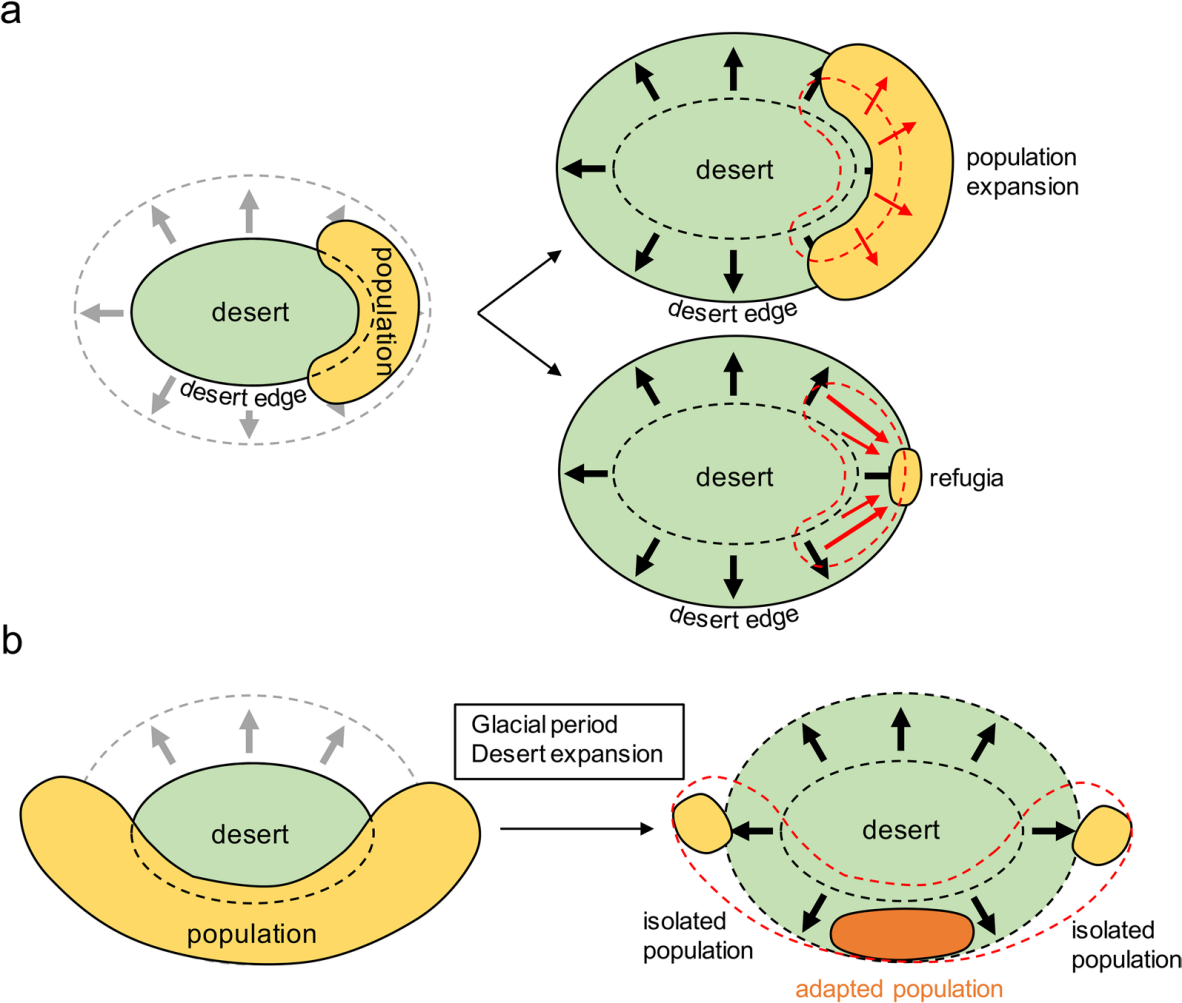
Effects of global climate change since the Quaternary in northwestern China on plant diversity and distribution. **a** Higher genetic diversity was discovered at desert edges. With desert expansion, plant populations may migrate in the direction of expansion, resulting in an increase of rare alleles. Alternatively, populations may migrate to refugia, leading to the accumulation of previously isolated lineages and increased genetic diversity. **b** Outcome of geographical isolation caused by desert expansion. During desert expansion, decreased moisture and strong winds may act as barriers to habitats and strengthen population isolation. With decreased gene flow from other populations, beneficial alleles could be more easily fixed, accelerating adaptation to arid habitats and causing genetic and ecological differentiation among populations

Biogeographic studies have increasingly shown how climate-induced aridification contributes to speciation, with several different possible effects. First, desert-adapted populations can expand or contract their distribution with the expansion of desert areas (Fig. 1a). During desert expansion, populations often respond through migration into newly suitable habitats at the desert’s margins, facilitated by rare allele accumulation, which results in increased genetic diversity (Jiang et al. 2019). Founder-flush processes at desert edges preserve rare alleles and promote the fixation of advantageous ones, such as drought-tolerant genes, by reducing drift through rapid population growth (Slatkin 1996). Alternatively, populations may retreat to isolated refugia during desert expansion, which may maintain genetic diversity through lineage segregation. For instance, *Lagochilus ilicifolius*, found in northern China and parts of Mongolia and Russia, demonstrated substantially high genetic diversity through chloroplast DNA analyses (Ma et al. 2012; Meng et al. 2015). This higher cpDNA haplotype diversity in desert edge regions suggests that diversification occurred along the expansion of deserts, or desert margins acted as refugia during harsh climates, which preserved the adaptive alleles for further ecological expansion.

A second possibility is that the desert expansion disrupts gene flow between established populations, leading to isolated populations and eventually speciation (Fig. 1b). For example, desert expansion during the Quaternary glacial stages in China was linked to weakened winds, enhanced atmospheric subsidence, and reduced moisture transport, all of which disrupted biota migration (Bush et al. 2004). This intensified aridification and large-scale desert expansion in northwestern China significantly altered the region’s hydrology and climate. As desert expansion fragmented habitats, isolated populations experienced reduced gene flow, intensifying founder-flush effects. Founder-flush effects and drift during isolation accelerate local adaptation and the fixation of beneficial alleles under arid conditions (Slatkin 1996). This emphasizes the evolutionary importance of marginal populations, which may develop crucial local adaptations for survival amid future climate changes. Additionally, a strong negative disequilibrium between low-frequency alleles at linked loci may serve as evidence of these founder-flush dynamics (Slatkin 1996), emphasizing their potential role in population differentiation and survival in extreme environments. The historical dynamics of desert expansion resulted in fragmented habitats, which likely impacted the genetic structure of drought-tolerant species. These processes provide insight into how populations, such as desert shrubs like *A. mongolicus*, developed unique adaptations throughout their range.

### Biogeographical characteristics of northwestern China and its desert plants

Northwestern China’s complex topography and arid climate create a unique biogeographic setting for desert plant diversification (Guo et al. 2002; Meng and Zhang 2013; Sun et al. 2010). Most plant biogeographical studies focus on the Sino-Japanese Floristic Region, known for its high temperate biodiversity and as a refuge for Tertiary relict species during Quaternary glacial cycles (Myers et al. 2000), as well as on endangered or endemic species in the Hengduan Mountains and the Qinghai-Tibet Plateau (QTP) (Qiu et al. 2011). In contrast, the effects of Quaternary climate changes on species in northwestern China’s arid regions are less understood, with limited knowledge of drought-adapted plant evolutionary histories. Species dynamics in these regions likely differ, as increased aridity rather than coverage of ice sheets may have strongly influenced plants here during glacial periods (Guo et al. 2002; Sun et al. 2010).

Northwestern China’s arid climate and varied topography, comprising mountains and deserts, foster distinct ecosystems (John et al. 2013; Yang et al. 2004; Zhu et al. 2019). This region is part of Asiatic Desert Subkingdom, far from marine influences, resulting in minimal moisture from air masses (Wei and Wang 2013; Yao et al. 2021). The QTP uplift has also diminished westerly winds, establishing an arid climate center in Eurasia (Yunfa et al. 2011). Drought-resistant vegetation, including shrubs and herbaceous plants from families like Chenopodiaceae, Lamiaceae, and Fabaceae, dominates this area (Meng et al. 2015). Conversely, the Eurasian Forest Subkingdom includes regions such as the Altai and Tianshan Mountains, which have experienced glaciation and climate shifts during the Quaternary period (Lehmkuhl and Owen 2005; Xu et al. 2010; Yi et al. 2004). These regions are characterized by cold-temperate and temperate montane coniferous forests featuring species from families like Pinaceae, Cupressaceae, and Betulaceae (Meng et al. 2015). These unique and extreme settings provide a natural laboratory to study the evolution and acclimation of desert plants. The unique biogeographical features of northwestern China influenced the distribution and isolation of desert plant populations, highlighting the importance of examining genetic differentiation and local adaptation in these plants.

### The QTP uplift and historical climate changes shaped the diversity of desert plants in northwestern China

Desert plants in northwestern China exhibit striking biogeographic patterns shaped by glacial-interglacial cycles that drove range shifts, isolation, and genetic divergence (Jia and Zhang 2019; Nobuko et al. 2011; Shahzad et al. 2017; Shi and Zhang 2015; Yisilam et al. 2022). As the climate cooled and became more arid, these plants likely migrated to warmer, more humid habitats, increasing genetic and species diversity. The QTP uplift since the Miocene intensified inland aridity by blocking moist air, accelerating desert formation (Clark et al. 2005; Fang et al. 2005; Wang et al. 2008; Zhenhan et al. 2008). Insufficient precipitation accelerated the formation of deserts, the Gobi, and loess deposits (Guo et al. 2002), leading to the expansion of major deserts, including the Taklamakan Desert in the Tarim Basin, the Gurbantünggüt Desert in the Junggar Basin, and the Badain Jaran-Tengger Desert north of the Hexi Corridor (Yang et al. 2004, 2011).

Studies suggest that during the Quaternary climatic fluctuations, desert plants experienced multiple migrations and divergences, fostering both intra- and interspecific genetic differentiation, for example, *Amygdalus mongolica* (Zhang et al. 2022), *Haloxylon ammodendron* (Chen et al. 2022), etc. The cooling during the Pleistocene increased desert aridity in China (Ding et al. 2005; Fang et al. 2002). Pleistocene orogeny in the Tianshan Mountains and adjacent ranges created rain shadows (regions with reduced precipitation, such as leeward slopes) that have promoted population isolation and divergence (Sun and Zhang 2009). High- to mid-latitude species faced ice sheets during glacial periods, while low-latitude regions experienced extreme aridity and lower temperatures (Willis et al. 2004). These climatic conditions expanded deserts as the Gobi, fragmenting habitats of arid-zone species and facilitating allopatric divergence, which may lead to speciation.

**Fig. 2:**
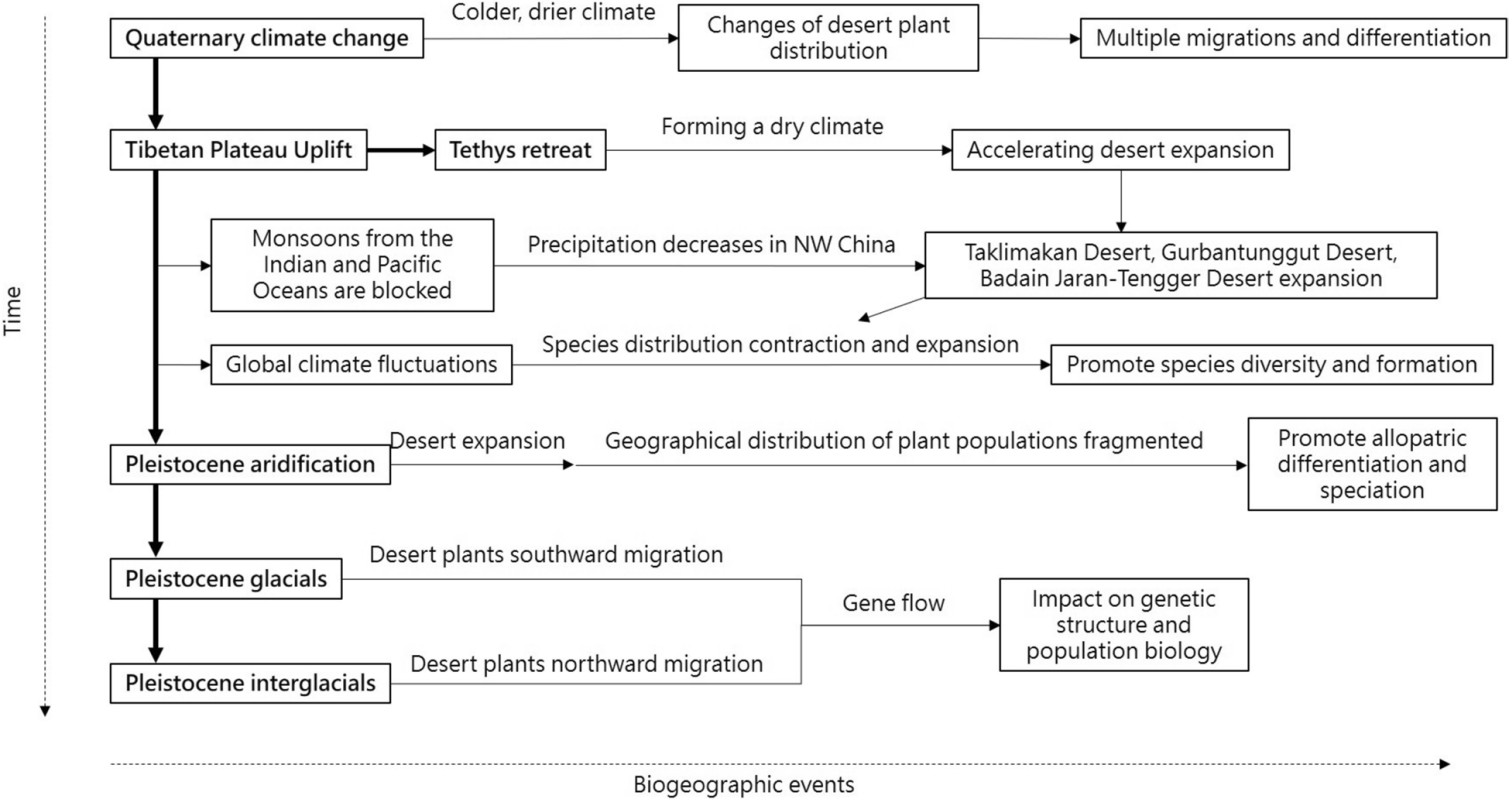
The changes in biogeographic process in northwestern China caused by global climate change since the Quaternary

The desert plants in the Hexi Corridor exhibit notable biogeographic characteristics, including high regional differentiation and genetic variation among populations due to geographic isolation and environmental selective pressures (Chen et al. 2009; Ge et al. 2005; Jiang et al. 2019). These desert plants have developed adaptive strategies in response to climatic changes with glacial-interglacial cycles leading to range expansion and contraction, enabling drought-adapted plants to thrive under changing aridity. For instance, a biogeographic study of *Gymnocarpos przewalskii* illustratehow geographic isolation and climatic oscillations have contributed to genetic differentiation among populations (Ma and Zhang 2012). Geographic barriers also have driven divergence and speciation in desert species, with plants like *Lagochilus ilicifolius* exhibiting migrations southward during glacial periods and northward during interglacial periods (Meng and Zhang 2011). The QTP uplift intensified the winter monsoon, causing cooling in the mid- to high-latitude interiors of Asia during the late Quaternary. This led to drier conditions that expanded deserts in northern China, which shaped the divergence of *L. ilicifolius* during the middle Pleistocene (Meng and Zhang 2011) (Fig. 2).

Notably, marginal populations surviving during ecological contractions often face intense selective pressures and isolation, leading to adaptive divergence (Su et al. 2016). These populations may hold unique genetic variants that improve their resilience, emphasizing their importance in coping with long-term climate change. Ancestral populations of *Ammopiptanthus* in the Alxa Desert (*A. mongolicus*) and the Tianshan Mountains (*A. nanus*) demonstrate considerable genetic differentiation, indicating their prolonged geographic isolation and specialized adaptation to changing climates (Su et al. 2016).

Taken together, the biogeographic histories of desert plants highlight that their genetic structures are shaped by historical isolation, range shifts, and survival strategies in varied habitats. These environments, particularly the desert margins, have acted as refugia or corridors during climate changes, supporting unique genetic variants. Understanding their adaptations is essential for assessing species resilience to ongoing climate change and its effect on conservation efforts. The uplift-driven climatic changes not only diversified desert flora but also shaped the adaptive potential of populations inhabiting contrasting environments. Recognizing this historical influence is essential for interpreting the ecological and genetic adaptations highlighted in Sect. "Ecological and genetic adaptations of plants to arid climates: a case from the evergreen shrub Ammopiptanthus mongolicus".

## Ecological and genetic adaptations of plants to arid climates: a case from the evergreen shrub *Ammopiptanthus mongolicus*

*Ammopiptanthus mongolicus*, a drought-adapted evergreen shrub and nationally protected species, exhibits ecological, physiological, and genomic adaptations to arid environments (Liu et al. 2001; Yang et al. 2022a). It is primarily distributed across low-to-middle elevations in northern Ningxia, northwestern Shaanxi, northeastern Gansu, and Wuhai City in Inner Mongolia, spanning between 102°-110° E longitude and 37°-43° N latitude (Liu et al. 2001). As a relic genus dating back to the early Miocene (Xie and Yang 2012), its ancestors are thought to have been among the xerophytic species of the Tethyan flora (Liu 1995; Sun 2002; Sun and Li 2003). These plants likely dispersed to the Alxa Desert through the Hexi Corridor (Liu 1995). Approximately 770,000 years ago, extreme climatic fluctuations reduced and isolated suitable habitats, resulting in allopatric divergence (Su et al. 2016). The diversification and speciation of *Ammopiptanthus* may have resulted from a series of geological events, including geographic isolation triggered by the formation of the Arctic ice sheet and the QTP uplift, which drove desertification and aridification in Central Asia (Ge et al. 2005).

The desertification of Central Asia significantly led to the extinction of most Tertiary plant species and the survival of a few relict species in limited refugia (Su et al. 2016), which likely occurred in *A. mongolicus*. Studies indicate that the nuclear genome of *A. mongolicus* comprises two major lineages, one distributed in the Tengger Desert and the other in the Ulan Buh Desert and Helan Mountains (Chen et al. 2009). Climatic oscillations during the late Quaternary are thought to have played a pivotal role in shaping the biogeographic distribution and genetic structure of *A. mongolicus* (Hewitt 2004). For instance, ^14^C analyses indicate that the Ulan Buh Desert was a large lake during the early Holocene, and the current distribution of *A. mongolicus* may reflect rapid expansion from nearby refugia (Jia and Yin 2004). The following subsections present evidence from its ecological distribution, dispersal strategies, physiological traits, and molecular adaptations.

### Physiological adaptation of *A. mongolicus* to drought and extreme environmental stress

To cope with diverse environmental stresses in arid and extreme habitats, *A. mongolicus* exhibits a suite of integrated physiological and biochemical adaptations that ensure survival, stress tolerance, and reproductive success (Shen et al. 2015; Zhu et al. 2020). The root system of *A. mongolicus* exhibits a dual structure, featuring both shallow and deep roots, enabling the plant to utilize water from surface soil and groundwater, respectively (Zhu et al. 2020). During the growing season, groundwater contributes approximately 49.6–57.4% of the plant’s water intake (Zhu et al. 2020). Moreover, compared to other deciduous shrubs, the leaves of *A. mongolicus* exhibit higher stable carbon isotope values, indicating a higher water use efficiency (WUE) under prolonged arid conditions (Zhu et al. 2020).

A comparison of *A. mongolicus* and *Zygophyllum xanthoxylon* under saline-alkaline and drought stress during seed germination and seedling growth showed that *A. mongolicus* completely inhibited germination under high stress, whereas *Z. xanthoxylon* exhibited a higher germination rate and shorter germination time (Shen et al. 2015). Salinity had a more significant negative impact on germination rate and seedling growth than drought, suggesting that while both species inhabit arid environments, drought stress adversely affects the germination and seedling stages of *A. mongolicus*. This supports the hypothesis that *A. mongolicus* is drought-tolerant but not drought-preferring (Liu and Qiu 1982). Under low temperatures, *A. mongolicus* prevents photooxidative damage while experiencing a significant decline in photosynthetic capacity (Yang et al. 2022b). Low temperatures inhibit the Calvin cycle, while high temperatures induce oxidative stress responses via metabolic gene regulation, demonstrating strong resilience (Yang et al. 2022b).

The thick cuticle on *A. mongolicus* leaves (up to 18 μm) reduces water loss and comprises long-chain fatty acids synthesized by the fatty acid elongase complex (Dimopoulos et al. 2020; Han and Li 1992). Aside from phenotypic adaptation, Zheng et al. (2023) conducted multiscale analyses, including leaf cell wall extractomics (leaf transcriptome, apoplast proteome, and apoplast metabolome), and found significant seasonal accumulation of PR3 and PR5 family proteins during autumn and winter. These proteins likely enhance frost tolerance through antifreeze activity, highlighting the importance of cell wall metabolism adjustments, calcium signaling, and MAPK cascade responses in *A. mongolicus*’s frost resistance (Zheng et al. 2023). The eco-chemical stoichiometry of different *A. mongolicus* organs show distinct patterns (Dong et al. 2023; Yang et al. 2020). Leaf nutrient patterns (high C and N, low P and K) support drought defense, while enriched N, P, and K in flowers and seeds ensure reproductive success (Dong et al. 2023). These differences in nutrient allocation among organs reflect *A. mongolicus*’s adaptive responses to leaf cells. These physiological responses are potentially crucial for adapting to the local environment and are closely linked to the species’ dispersal and colonization strategies.

### Dispersal and pollination strategies in *A. mongolicus* in arid environments

The seed dispersal strategy of *A. mongolicus* enables it to colonize isolated desert patches and maintain population persistence. The genetic structure of *A. mongolicus* populations is often assumed to follow the isolation-by-distance (IBD) model (Ge et al. 2005). However, increasing evidence suggests that geographic distance or barriers alone do not fully explain gene flow patterns; environmental differences also play a critical role (Sexton et al. 2014; Shafer and Wolf 2013; Wang and Bradburd 2014). Selective pressures can drive population differentiation more rapidly than genetic drift, even over small geographic scales. Research has shown that adaptive divergence due to environmental heterogeneity better explains population differentiation in *A. mongolicus* than IBD (Jiang et al. 2019), highlighting the importance of selective processes over neutral ones.

The seed pods of *A. mongolicus* exhibit three main morphological types: indehiscent, dehiscent, and twisted pods (Thiede and Augspurger 1996). Indehiscent pods are typically heavier, have higher water content, and are adapted for early abscission with prolonged dormancy, allowing seeds to await favorable germination conditions. Dehiscent and twisted pods are lighter and suited for long-distance dispersal, reducing the pressures of intraspecific competition (Thiede and Augspurger 1996). This morphological diversity provides a balance between strategies to adapt to different environments: indehiscent pods lower predation risk, while dehiscent pods enhance long-distance dispersal capacity, forming a risk-hedging strategy against fluctuating environments (Arshad et al. 2019; Lu et al. 2015; Villa Martín et al. 2019).

*Ammopiptanthus mongolicus* primarily relies on insect pollination, with major pollinators including species of *Anthophora* (e.g., *A. uljanini* and *A. fulvitarsis*), *Chalicodoa deserticola*, and *Hoplitis alashanica* (Liu 2006). Seed dispersal is predominantly gravity-mediated, with the distance of gene flow constrained by the limited flight range of pollinators. Precipitation is a key factor influencing seed dispersal and germination in *A. mongolicus* (Jiang et al. 2019). Dry air conditions favor long-distance dispersal, while humidity affects seed dormancy duration and survival rates (Baskin et al. 2014; Zhao et al. 2009). Studies have shown that seed dispersal distances increase under arid conditions, but humidity variations can significantly impact seed survival and germination success (Baskin et al. 2014; Zhao et al. 2009).

### Genomic insights into the arid adaptation of *Ammopiptanthus*

Molecular analyses reveal a suite of stress-responsive genes that underlie the environmental resilience of *Ammopiptanthus* species (Feng et al. 2024; Gao et al. 2018). The first whole-genome sequencing of the genus *Ammopiptanthus* was conducted on *A. nanus* in Xinjiang, using PacBio sequencing (Gao et al. 2018). An 823.74 Mb genome with an N50 of 2.76 Mb was assembled, and 74.08% of the genome consisted of repetitive elements, predominantly long terminal repeats (LTRs), was found. A total of 37,144 protein-coding genes were annotated, 96.71% of which had putative functions. After, Feng et al. (2024) achieved chromosome-level assembly of *A. mongolicus* (843.07 Mb) using PacBio, Illumina, Bionano optical mapping, and Hi-C technologies. Repetitive elements accounted for 70.71% of the genome, with LTR retrotransposons (Ty1/Copia and Ty3/Gypsy) dominating. Annotation revealed 47,611 protein-coding genes. Unlike other legumes, *Ammopiptanthus* has not experienced recent whole-genome duplication since 580,000 years ago. Gene family expansions associated with stress response and metabolic pathways also suggest adaptive evolution to arid environments (Feng et al. 2024).

Transcriptome analysis identified drought-responsive genes such as *KCR*, *WSD1*, *CER3*, and *LTPs*, which are highly expressed in leaves and upregulated under dehydration stress (Feng et al. 2024). Additionally, genes involved in ethylene (*ACS*, *ACO*, *ETR*, *EIN3*, *ERF*), abscisic acid (ABA), and jasmonic acid (JA) pathways were significantly induced under stress, highlighting their roles in dehydration tolerance. Heterologous expression of the *AmERF2* gene in *Arabidopsis* enhanced drought resistance, emphasizing ethylene’s importance in stress adaptation (Feng et al. 2024). In addition, several drought-tolerant and resistant genes in *A. mongolicus* and *A. nanus* enhance root growth, water retention, osmolyte accumulation, and oxidative stress regulation under drought stress. Functional validation through transgenic *Arabidopsis thaliana* experiments confirms their roles in enhancing drought resilience and adaptation (Table 1).

**Table 1:** Drought-tolerant and drought-resistant genes identified in *Ammopiptanthus mongolicus* and *A. nanus*

Species	Gene	Function	Citation
*A. mongolicus*	*AmDREB2C*	Enhancing germination rate, root growth, biomass accumulation, and water hold capacity under osmotic stress	Yin et al., (2018)
	*AmDHNs*	Promoting plant root growth and leaf growth under osmotic stress	Cui et al., (2020)
	*AmERF2*	Increasing ethylene content	Feng et al., (2024)
	*AmNAC11*	Promoting plant root growth at the germination stage	Pang et al., (2019)
	*AmNAC24*	Reducing malondialdehyde content, and promoting proline accumulation	Dorjee et al., (2024)
	*AmNHX2*	Enhancing water-retaining capability under drought stress	Wei et al., (2011)
	*AmVP1*	Accumulating more sodium and potassium in their leaves after salt stress, and retaining more water while producing less malondialdehyde during drought stress	Wei et al., (2012)
*A. nanus*	*AnDHN*	Causing increased germination rate, higher relative water content, higher proline content, increased peroxidase and catalase activities, lower contents of malondialdehyde, H2O2 and O2–, and longer root length	Sun et al., (2021)
	*AnLEA30*	Causing lower relative electrolyte leakage and malondialdehyde	Liu et al., (2024)
	*AnSAUR50*	Negatively regulating the root’s growth and stomatal closure under drought stress	Zhang et al., (2024)
	*AnVP1*	Enhancing individual growth, root growth, ion accumulation, and proline content, and lowering malondialdehyde content and relative electrolytic leakage	Yu et al., (2017)
	*AnWRKY29*	Improving drought tolerance by increasing the uptake of inorganic salt ions under water stress	Wang et al., (2023)
	*AnWRKY40*	Interfering with the ROS-scavenging pathway and osmolyte accumulation process	Hao et al., (2020)

Gene functions are confirmed by transgenic experiments in *Arabidopsis thaliana* and drought stress assays. Only genes validated through transgenic approaches are listed; those supported solely by transcriptomic or expression analyses are excluded

Molecular mechanisms underlying adaptation to drought and low temperatures in *A. mongolicus* have been revealed, identifying thousands of genes and multiple stress-related metabolic and signaling pathways (Gao et al. 2015; Wu et al. 2014). Among these, flavonoids and membrane proteins play key roles in stress tolerance, while chloroplasts enhance cold resistance by maintaining photosynthetic efficiency. Transcription factors such as the AP2/EREBP, NAC, WRKY, and bHLH families are involved in stress signal transduction, shedding light on the genetic mechanisms of *A. mongolicus* adaptation to extreme environments (Wu et al. 2014). These molecular adaptations not only underscore the evolutionary and conservation significance of *A. mongolicus* but also provide a genetic basis for population-level comparisons across its distribution range, enabling assessment of how variation in these stress-responsive pathways contributes to differential resilience to climate change.

### Adaptive strategies in marginal and core populations of *A. mongolicus* under climate change

Populations of *A. mongolicus* show distinct genetic differences between core and marginal regions (Yang et al. 2022a). Core populations, found in the central Alxa Desert, exhibit higher genetic diversity due to larger effective population sizes and stable environments. In contrast, marginal populations at ecological limits experience founder-flush processes, leading to increased genetic differentiation and unique variants not present in core populations. These variants may be adaptations to extreme conditions, indicated by higher *F*_ST_ values (*F*_ST_ = 0.136 ± 0.033 between core and adaptive marginal populations, while *F*_ST_ = 0.082 ± 0.035 between core and non-adaptive marginal populations) and specific environmental loci (cold- and drought-stress associated genes) (Yang et al. 2022a). Additionally, intense selection in marginal habitats can fix beneficial alleles but may also increase genetic load from smaller population sizes and inbreeding (Yang et al. 2025). Overall, core populations provide genetic stability, while marginal populations act as hotspots for adaptation to environmental changes.

**Fig. 3:**
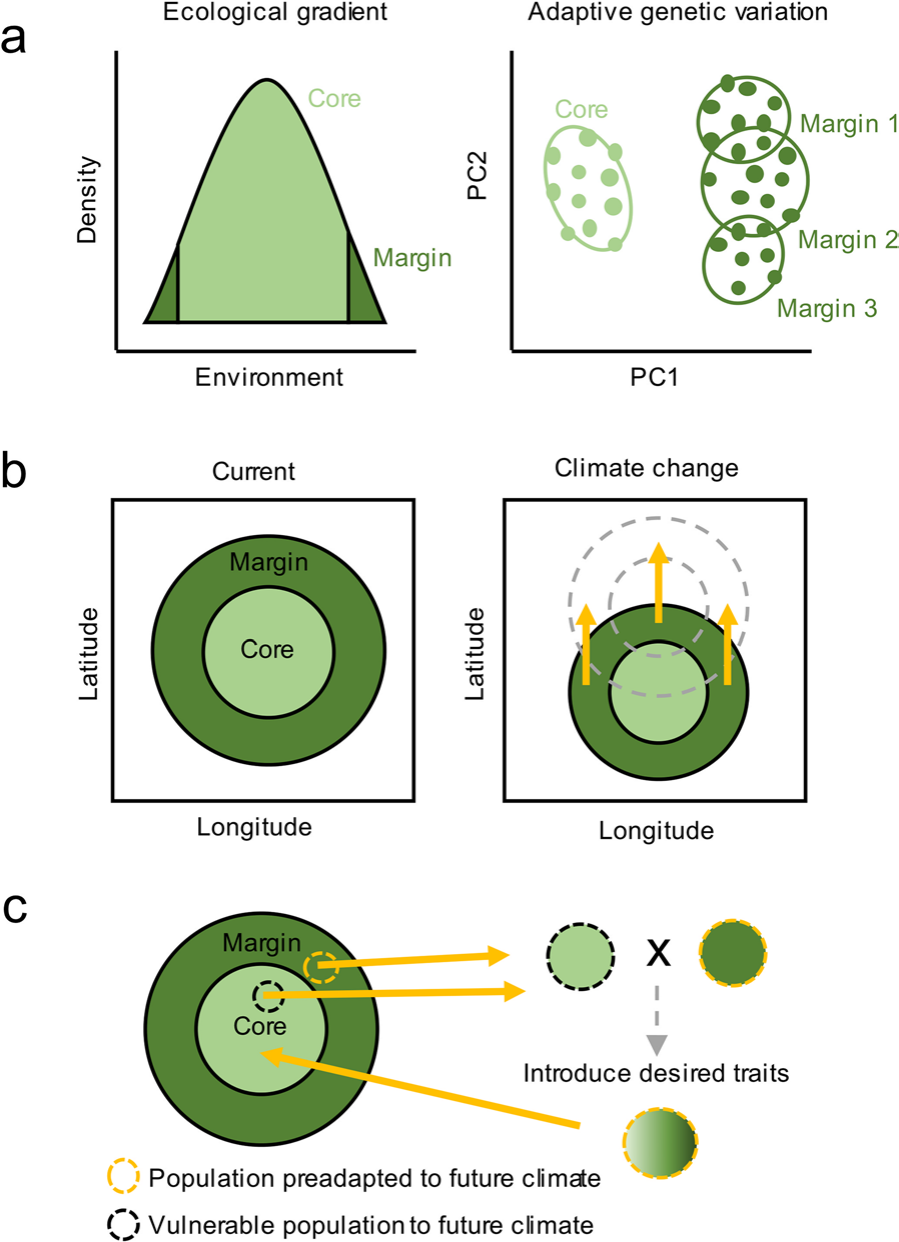
Climate change affects diversity and distribution of core and marginal populations. **a** Ecological and adaptive genetic variation among core (light areas) and marginal (dark areas) populations. Core populations occupy optimal habitats, while marginal populations are mostly found in extreme regions. Unlike core populations, multiple marginal populations may demonstrate parallel adaptation to environmental pressures and form a continuous group that is genetically differentiated from the core. **b** Climate-change-induced migration. It is assumed that populations will move northward in response to a warming future climate. Core and marginal populations may interact with previously isolated populations, enhancing gene flow between core and marginal populations to exchange beneficial alleles or impeding the fixation of adaptive alleles. **c** Example of assisted gene flow. To avoid the stochasticity of gene flow and geographical limitations, assisted migration may be an approach to increase local resilience. After selecting the source (preadapted individuals) and the sink (vulnerable individuals), individuals with desired traits or adaptive alleles can be artificially introduced into the gene pool of the sink population

The core populations of *A. mongolicus* are in stable, relatively moderate environments with larger populations and higher genetic diversity. In contrast, marginal populations inhabit the species’ distribution periphery, where environmental conditions are more variable and extreme (Fig. 3a), such as low winter precipitation and significant seasonal temperature fluctuations (Carvalho et al. 2019). Marginal populations must adapt to harsher conditions, including extreme cold, drought, and nutrient-poor soils, which challenge their survival and growth (de Lafontaine et al. 2018; Ginwal et al. 2024).

Previous climatic analysis reveals significant environmental differences between northern and southern populations of *A. mongolicus* (Yang et al. 2022a, 2025). Core populations in regions such as western Inner Mongolia and northwestern Gansu experience moderate temperatures, smaller seasonal temperature ranges, and stable precipitation during the wettest season, creating optimal conditions for growth and maintaining genetic diversity. Conversely, marginal populations in eastern Xinjiang and the high-altitude deserts of Ningxia endure extreme temperatures, low precipitation, and harsh winters, requiring unique adaptations to survive (Yang et al. 2022a).

Marginal populations, particularly those in climate-edge regions, are considered to face heightened risks of habitat deterioration or local extinction due to harsher environmental conditions (Aitken et al. 2008; Gougherty et al. 2021). However, marginal populations often exhibit local adaptations, such as enhanced drought and cold tolerance, due to prolonged exposure to environmental stressors (Chuang and Peterson 2016; Leger et al. 2009; Volis et al. 2016). While founder and bottleneck effects reduce genetic diversity, these populations benefit from de novo mutations, which can surf along standing genetic variation to optimize adaptation to marginal conditions (Yang et al. 2022a). This is evident in the fact that the core populations of *A. mongolicus* have higher genetic diversity (*H*_*e*_ = 0.179 ± 0.023, π = 0.189 ± 0.023), whereas the marginal populations have less (*H*_*e*_ = 0.157 ± 0.017, π = 0.165 ± 0.019). However, new adaptive changes in the marginal populations may enhance their genetic diversity (*H*_*e*_ = 0.179 ± 0.009, π = 0.187 ± 0.009) (Yang et al. 2022a). These marginal populations adapt to cold and drought via overlapping gene functions, with pleiotropy playing a crucial role in simultaneously addressing multiple environmental pressures (Pang et al. 2013; Wu et al. 2014).

Physiological adaptations, such as the ability to withstand temperature fluctuations without shedding leaves, may be critical for *A. mongolicus*, given its evergreen shrub form. Marginal populations rely on maintaining and fixing beneficial mutations that confer advantages across diverse stressors, creating parallel adaptive patterns among geographically isolated populations. Relaxed selective constraints, genetic drift, and limited gene flow further shape their unique genetic characteristics (Yang et al. 2022a).

### The role of marginal populations in the conservation of *A. mongolicus* under climate change

Marginal populations, though often small and isolated, are essential to the long-term evolutionary potential of *A. mongolicus* under climate change, as they harbour unique genetic variants that contribute to adaptation and species resilience (Fig. 3b). According to Sobel et al. (2010), varying habitat conditions can accelerate population divergence due to the necessity to adapt to distinct ecological pressures. Marginal populations exposed to prolonged extreme conditions may experience increased genetic load (Takou et al. 2021) due to the accumulation of deleterious alleles (Sachdeva et al. 2022). However, these conditions can also drive adaptation and enable populations to cope with rapid environmental changes (Hoffmann and Sgrò, 2011).

Intrinsic genetic factors, such as mutation accumulation, may have a greater impact on adaptation than previously recognized, sometimes surpassing the effects of environmental changes. Wili (2019) highlighted that mutation accumulation significantly limits niche expansion, especially in small or isolated marginal populations, resulting in reduced population growth rates and increased genetic load. These findings challenge the traditional view that environmental pressures are the primary drivers of adaptation, emphasizing the importance of genetic factors such as mutation load and genetic diversity in shaping species distributions. This underscores the need to integrate genetic considerations into conservation and management strategies (Savolainen et al. 2013).

Gene flow can introduce novel genetic variants that reduce the detrimental effects of genomic load and enhance adaptation to extreme environments, particularly in marginal populations that often face unpredictable conditions (Buckley et al. 2021; Fitzpatrick and Reid 2019; Sachdeva et al. 2022). By promoting adaptive traits across populations, gene flow supports resilience and survival in diverse ecological settings, particularly for populations in extreme environments. Assisted gene flow can further enhance genetic diversity and adaptive potential by facilitating genetic rescue by introducing adaptive traits from marginal populations (Aitken and Whitlock 2013).

Future genetic adaptations in *A. mongolicus* are likely to reflect environmental factors such as summer heat, winter rainfall, and monthly precipitation variability rather than winter temperature and summer rainfall alone (Yang et al. 2025). Marginal populations may serve as key reservoirs of adaptive diversity amid accelerated climate change (Lenormand 2002). Previous findings also suggest that marginal populations harbor more multifunctional adaptive genes, reinforcing their role as critical repositories of genetic resources (Yang et al. 2025). Therefore, marginal populations should be treated with higher conservation priorities when genomic data is accessible and adaptive alleles are enriched.

In conclusion, conserving or even enhancing genetic diversity in marginal populations is vital for mitigating the impacts of environmental change. Maintaining gene flow among populations helps to preserve adaptive potential, particularly in extreme and rapidly changing environments (Jump and Peñuelas 2005). This highlights the importance of prioritizing conservation efforts for marginal populations, given their higher risk of maladaptation and their key role in supporting species-wide resilience.

## Conclusion and conservation perspective

The conservation of desert-adapted species, such as *A. mongolicus*, accentuates the significance of genetic strategies in arid environments. Marginal populations, limited by founder effects and environmental stress, possess critical adaptive traits that enable survival in extreme conditions like drought and cold (Hoffmann and Sgrò, 2011; Lenormand 2002). They act as vital genetic reservoirs, providing valuable alleles that enhance species-wide resilience to climate change (Fig. 3b) (Fady et al. 2016). However, habitat fragmentation and increased genetic load pose risks that require targeted conservation efforts. Maintaining gene flow between core and marginal populations can help reduce genetic load and facilitate the spread of adaptive traits throughout the species’ range (Angert et al. 2020). Considering the limited migration abilities of *A. mongolicus*, assisted gene flow may also be a potential approach to increase diversity and resilience to future climate (Fig. 3c). For plants in arid regions, conservation strategies should prioritize habitat preservation, assisted gene flow, and adaptive genetic management (Yang et al. 2025). In the case of *A. mongolicus*, its dependence on both physiological resilience and genetic diversity highlights the importance of marginal populations as sites of evolutionary potential (Yang et al. 2022a). Protecting these populations not only ensures the species’ adaptive capacity but also serves as a model for managing other desert flora facing similar challenges in a rapidly changing climate.

## Data Availability

Not applicable.
